# Computational identification of potential inhibitors for chikungunya virus nsP2pro

**DOI:** 10.1098/rsos.252347

**Published:** 2026-10-07

**Authors:** Hoang Anh Nguyen, Son Tung Ngo

**Affiliations:** ^1^ NTT Hi-Tech Institute, Nguyen Tat Thanh University, Ho Chi Minh City, Vietnam; ^2^ Nguyen Tat Thanh University Center of Hi-Tech Development, Saigon Hi-Tech Park, Ho Chi Minh City, Vietnam; ^3^ Laboratory of Biophysics, Institute for Advanced Study in Technology, Ton Duc Thang University, Ho Chi Minh City, Vietnam; ^4^ Faculty of Pharmacy, Ton Duc Thang University, Ho Chi Minh City, Vietnam

**Keywords:** chikungunya virus, non-structural protein 2 protease, docking, free energy perturbation, molecular dynamics simulation

## Abstract

Chikungunya virus remains a crucial global health issue with no approved therapy available. In particular, the viral replication is associated with the non-structural protein 2 protease (nsP2pro). In this study, possible inhibitors for nsP2pro from the National Cancer Institute (NCI) database were screened via computational approaches. Molecular docking simulations were initially predicted top potential ligands. MD simulations were then performed to refine the ligand binding pose. During the equilibrium intervals, the ligands formed rigid contacts with the residues Asn476, Ala511, Tyr512, Tyr544 and Trp549 of nsP2pro. Moreover, five compounds probably prevent the biological activity of nsP2pro by increasing the distance of Cys478-Sγ–Nε-His548 and Ser482-Oγ–Nε-His548 pairs. Furthermore, perturbation simulations finally confirmed that NSC 319990, NSC 80731, NSC 80735, NSC 67436 and NSC 37553 compounds exhibited significantly stronger binding affinity, ranging from –9.70 ± 0.61 to –11.71 ± 1.25 kcal mol^–1^, than that of the available inhibitor RA-0002034, Δ*G*_FEP_ = − 6.45 ± 0.32 kcal mol^–1^. Besides, the strong correlation between Δ*G*_FEP_ and Cys478-Sγ–Nε-His548 pair suggests that a stronger binding ligand better inhibits the biological activity of nsP2pro. Overall, these findings suggest that five NCI compounds are highly promising candidates for further experimental validation as novel nsP2pro inhibitors.

## 1. Introduction

Chikungunya virus (CHIKV) is an enveloped RNA virus belonging to the genus *Alphavirus* that causes a viral illness known as chikungunya fever, which is transmitted by mosquitoes [1,2]. Chikungunya fever rapidly spread across 16 countries and territories in the Americas, Africa, Asia and Europe with 240 000 cases and 90 deaths in 2025 [3]. The symptoms include fever, nausea, painful joints, fatigue and a rash [3]. The infection also causes heart damage [3] and influences the quality of life for up to six years [4,5]. The virus was first characterized in the early 1950s [1]. From then until now, this disease has seen multiple recurring outbreaks, yet a cure remains elusive.

CHIKV genome includes non-structural protein (nsP) 1 (negative-strand RNA synthesis), nsP2 (helicase and protease activity), nsP3 (RNA synthesis), nsP4 (RNA-dependent RNA polymerase), structural proteins C (capsid), E1-E3 (envelope glycoproteins) and 6K/TF (translation products of the same gene) [1]. Among these, nsP2 is a non-structural and multi-functional protein responsible for cleaving polyproteins into individual functional proteins and unwinding RNA for viral replication [6]. The enzyme includes two domains and one flexible linker. The first domain responds for unwinding viral RNA, a step necessary for replication. This domain also has NTPase and RTPase activity [7]. The methyltransferase-like and protease domain acts as molecular ‘scissors’, cleaving the viral polyproteins into individual functional proteins [8]. Thus, nsP2 becomes an attractive target for drug design [9–11], especially as there is no homologous enzyme, reducing the risk of toxicity.

CHIKV non-structural protein 2 protease (nsP2pro) is a flexible cysteine protease that can switch its catalytic mechanism, which poses both challenges and opportunities for drug design. Unlike typical cysteine proteases that rely on a single fixed Cys-His residue, CHIKV nsP2pro can use either a nearby Cys478 or Ser482 to catalyse the reaction [12–14]. Among these, in the primary nucleophile, the Cys478 is activated, which is typical of cysteine proteases. The Cys478 thiol group, after deprotonation by His548, breaks the peptide bond of the viral polyprotein, starting the cleavage process. Besides, if Cys478 is inhibited, Ser482 can take over the nucleophilic role. Ser482 hydroxyl group performs the same role of Cys478 thiol group, allowing the enzyme to remain active. This creates a backup catalytic mechanism. It may be argued that the successful inhibitor is required to be able to block both catalytic mechanisms of CHIKV nsP2pro. In addition, some critical residues of the protein can be listed as Ala475, Asn476, Trp479, Tyr512, Tyr544, Asn547, Trp549, Asn574 and Met707 [8,15].

The development of a viral therapy requires significant funding over a prolonged period using conventional approaches [16]. Currently, the application of computational methods to discover new potential inhibitors can significantly reduce the time and cost of drug exploration [17–20]. In particular, the ligand binding free energy can be characterized via atomistic simulations [21–23]. The inhibitory mechanism would then be illuminated within the computational investigation [24,25]. Moreover, several metrics of pharmacology and pharmacokinetics can be rapidly estimated using a web server or protocol [26–28]. Several approved drugs have been initially found using computational approaches, such as ritonavir for HIV-1 protease [29], zanamivir for neuraminidase [30] and nirmatrelvir for SARS-CoV-2 main protease [31].

In this work, the potential inhibitors for CHIKV nsP2pro were screened from the National Cancer Institute (NCI) Diversity Set VII database [32], with 1581 compounds clustering from almost 140 000 compounds. Initially, 1581 compounds were docked to the active site of CHIKV nsP2pro. A stronger docking affinity suggests a better potential inhibitor to the protease. The shortlist of these compounds was then refined via intensive molecular dynamics (MD) simulations. The binding mechanism of the potential inhibitors was also clarified over the conventional MD trajectories. Moreover, ligand binding affinity was confirmed via perturbation simulations.

## 2. Material and methods

### 2.1. Chikungunya virus non-structural protein 2 protease and ligand conformations

The three-dimensional structure of CHIKV nsP2pro was downloaded from the Protein Data Bank (PDB) with PDB ID: 4ZTB) [8]. In total, 1581 trial ligands were obtained from the NCI Diversity Set VII database [33]. Moreover, the three-dimensional structure of RA-0002034 [15], an available inhibitor, of nsP2pro was downloaded from the PubChem database [34] with the corresponding ID of 139018657.

### 2.2. Molecular docking simulation

AutoDock Vina [35] with altered empirical parameters [36] was employed to preliminarily investigate the ligand binding pose and affinity to CHIKV nsP2pro. Because 4ZTB contains no native inhibitor, the Venezuelan equine encephalitis virus (VEEV), an Alphavirus, nsP2pro with the PDB ID 8T8N [37] was employed to accurately characterize the CHIKV nsP2pro active site (cf. figure 1). Chain A of CHIKV nsP2pro, with sequence from 471 to 791, was selected as the docking receptor. The docking grid centre was chosen as centre of mass of the native ligand of VEEV nsP2pro after VEEV nsP2pro + its inhibitors were aligned to CHIKV nsP2pro via PyMOL tools [38]. The docking grid was selected as 20 × 20 × 20 Å, which is big enough to cover the whole of the binding site. The docking simulations were performed with an exhaustiveness value of 8, a setting statistically demonstrated to be sufficient for accurate pose prediction of standard organic ligands in comprehensive benchmarks [36,39]. Here, only one conformation with the strongest binding affinity was recorded.

**Figure 1.**
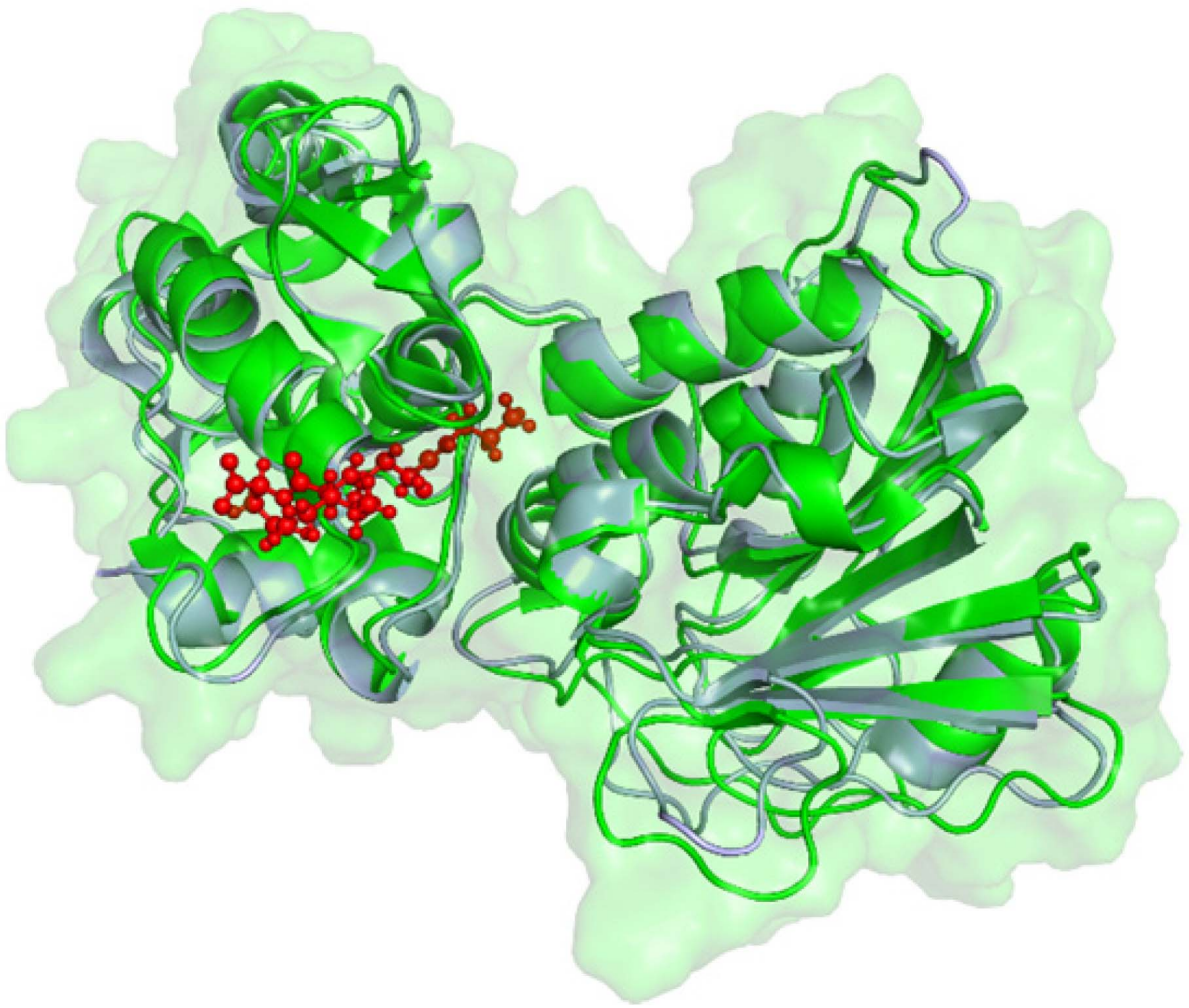
Conformation of CHIKV nsP2pro (green colour) in superposition state with VEEV nsP2pro (grey colour). In particular, the inhibitor of VEEV nsP2pro is denoted with red colour highlight.

### 2.3. Molecular dynamics simulation

GROMACS version 2023 [40] was employed to mimic two systems, including the CHIKV nsP2pro + inhibitor and the free inhibitor in solution. Particularly, nsP2pro and neutralized ions were topologized using the Amber99SB-iLDN force field [41]. Consequently, the TIP3P water model was employed to represent the water molecule [42]. The force field was used because it is the most suitable for free energy calculations [43,44]. Both systems—the nsP2pro + inhibitor and the free inhibitor—were inserted into a cubic periodic boundary condition box with a spacing of larger than 1.0 nm between system and boundary. The water molecules and neutralized ions were then inserted into the box. The number of atoms of the complex nsP2pro + inhibitor and the free inhibitor are *ca* 76 000 and 3660 atoms.

Both systems were initially minimized using the steepest descent method. Consequently, the nsP2pro + inhibitor and free inhibitor in solution were positionally constrained in canonical and isobaric-isothermal ensemble simulations of 100 ps each. The outcome conformation was used as the original structure for 200 and 20 ns of MD simulations at 310 K, corresponding to the complexed CHIKV nsP2pro + ligand and the free ligand in solution, respectively. During MD simulations, the coordinates of the solvated systems were recorded every 100 ps. The simulation was repeated three times for each system to guarantee the sampling statistics. In particular, the MD parameters were obtained from the previous studies [45,46]. The non-bonded contact between distinct atoms was updated within 0.9 nm every 30 fs. The electrostatic (cou) and van der Waals (vdW) interactions were calculated using particle-mesh Ewald and cut-off schemes within a range of 0.9 nm, respectively. Moreover, an accurate and efficient leap-frog stochastic dynamics integrator was employed to mimic the behaviour of the solvated systems. The V-rescale thermostat and Parrinello–Rahman extended-ensemble pressure coupling were used during MD simulations.

### 2.4. Perturbation simulation

The free energy perturbation (FEP) simulations can be used to determine the binding free energy between biomolecules with high accuracy [23,47,48]. In particular, during the simulations, the coupling parameter, λ, is used for modifying the systemic Hamiltonian. Thus, a molecule is removed from the solvated system. The difference in free energy change between the two states *available,* λ  =  0*,* and *removed,* λ  =  1*,* can be calculated via the Bennett's acceptance ratio approach [49]. The ligand binding free energy can be computed over perturbation simulations as the difference of free energy change in two processes including removing the inhibitor from CHIKV nsP2pro + inhibitor and the free inhibitor in solution. This approach is called the double-annihilation binding free energy method [50,51]. Moreover, in this work, the last snapshot of MD simulations was used as the initial conformation of perturbation simulations. The electrostatic coupling parameter, λ_cou_, was selected as 0.00, 0.10, 0.20, 0.35, 0.50, 0.65, 0.80 and 1.00. Furthermore, the vdW coupling parameter, λ_vdW_, was chosen as 0.00, 0.10, 0.25, 0.35, 0.50, 0.65, 0.75, 0.90 and 1.00. The value of the coupling parameters was chosen in line with the previous studies [23]. In addition, more details about the double-annihilation binding free energy calculations have been previously reported [23,52].

### 2.5. Analysis tools

Chemicalize, a tool of ChemAxon, was employed to estimate the inhibitor pKa [53]. A three-dimensional interaction diagram between CHIKV nsP2pro and its ligand was prepared using PLIP web application [54]. Intermolecular sidechain contact is calculated when the non-hydrogen spacing between distinct atoms is smaller than 0.45 nm. Intermolecular hydrogen bond (HB) contact is figured when two conditions are satisfied. The first condition is that the angle, ∠, between donor (D), hydrogen (H) and acceptor (A) must be larger than 135 degrees. The second condition is that the distance between D and A must be smaller than 0.35 nm. The non-hydrogen atoms root-mean-square deviation (RMSD) of the solvated system was calculated via GROMACS tools ‘gmx rms’. The distance between two atoms was computed via GROMACS tools ‘gmx mindist’. The clustering calculation was completed using ‘gmx cluster’ tools with a non-hydrogen atom cut-off of 0.3 nm. The absorption, distribution, metabolism and excretion (ADME) and chemical properties of compounds were estimated via pkCSM [55].

## 3. Results and discussion

### 3.1. Docking affinity and pose

As mentioned above, the CHIKV nsP2pro is one of the most favoured targets for drug design because it is associated with the viral replication [6]. In particular, the enzyme cleaves the polyproteins and unwinds the RNA. In particular, nsP2pro is a cysteine protease and it can recognize small peptide substrates. Notably, several investigations have characterized small compounds proficient of inhibiting CHIKV nsP2pro, such as RA-0002034 [15]. Besides, the structural compounds can be altered to improve their inhibitory and pharmacokinetic properties [56–58]. Therefore, screening the inhibitor for CHIKV nsP2pro from small compounds is a highly potential area of research. In this context, AutoDock Vina [35], an open-source molecular docking application, is one of the most popular protocols, with more than 28 000 citations to date. A modified version with altered empirical parameters, denoted as mVina, has a higher correlation coefficient, *R* = 0.62 ± 0.02, compared with the original version, *R* = 0.54 ± 0.02 [35,36]. The package was thus used to estimate the binding pose and affinity of 1581 NCI compounds to CHIKV nsP2pro. The distribution of the obtained molecular docking energy of these compounds to nsP2pro is shown in figure 2A. Specifically, the docking scores ranged from –4.9 to –13.3 kcal mol^–1^, with an average of −8.89 ± 1.42 kcal mol^–1^. The computed error was the standard deviation.

**Figure 2.**
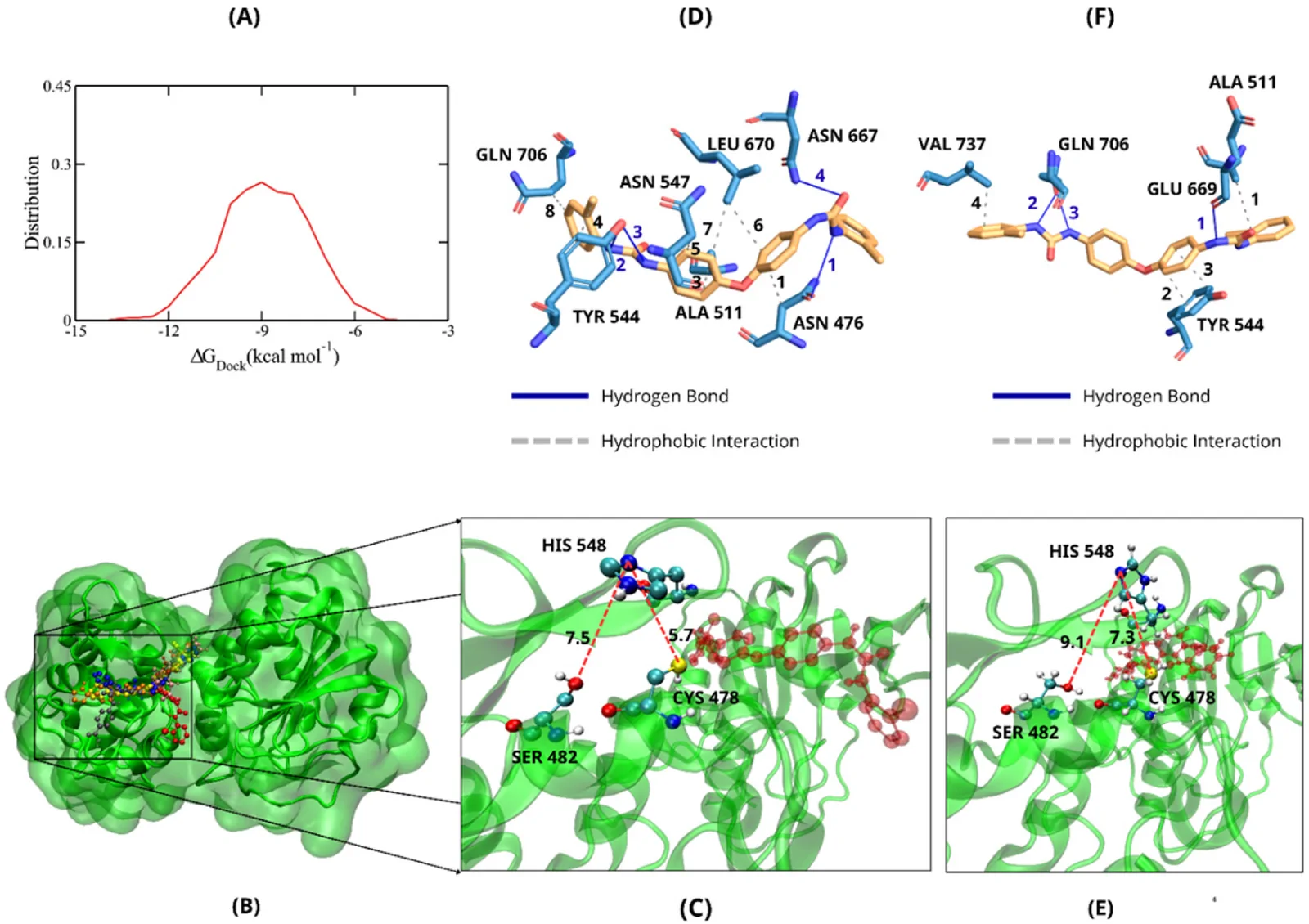
(A) Distribution of molecular docking results of 1581 NCI compounds to CHIKV nsP2pro. (B) The superposition of top-lead five NCI ligands and RA-0002034 in docking state with CHIKV nsP2pro. (C) Enlarged perspective of NSC 80731 binding to CHIKV nsP2pro active site, which is obtained via molecular docking simulations. In particular, the catalytic residues were denoted as Cys478, Ser482 and His548. Red dashed lines denote Cys478-Sγ–Nε-His548 and Ser482-Oγ–Nε-His548 pairs. (D) Interaction diagram between CHIKV nsP2pro and NSC 80731 in docking mode, which was prepared using PLIP web application [54]. (E) MD-refined binding pose of NSC 80731 to CHIKV nsP2pro and the pairs (red dashed lines) including Cys478-Sγ–Nε-His548 and Ser482-Oγ–Nε-His548. In particular, the MD-refined structure was obtained through clustering methods. (F) Interaction diagram between CHIKV nsP2pro and NSC 80731 in MD-refined binding mode, which was prepared using PLIP web application [54].

Five top-lead compounds with the strongest binding affinity to CHIKV nsP2pro are listed in table 1. Besides, RA-0002034 [15], an available inhibitor of nsP2pro, was also docked to the protease for comparison. The obtained result is also mentioned in table 1, in which the docking energy of –9.9 kcal mol^–1^ is in good agreement with the experimental value, Δ*G*_EXP_ = −9.27 kcal mol^–1^. The outcomes imply that mVina is an appropriate protocol for estimating the possible binding compounds to CHIKV nsP2pro. The binding pose between ligands and nsP2pro is shown in figure 2 and figures S1–S6 in the supplementary material. The docking interaction diagrams suggested that three residues—Tyr544, Asn547 and Leu670—may act as important elements during the ligand binding process because they form HB, hydrophobic or π-stacking contacts to CHIKV nsP2pro. The ligands may indirectly alter the pairs including Cys478-Sγ–Nε-His548 and Ser482-Oγ–Nε-His548, as they interact rigidly with the residue Asn547. It should be noted that these pairs are associated with the catalytic domain of the enzyme. Molecular docking outcome implies that five top-lead compounds may act as potential inhibitors for CHIKV nsP2pro.

**Table 1.** The ligand binding affinity provided by molecular docking simulations.^a^

no.	ligand	Δ*G*_Dock_	Δ*G*_EXP_^b^
1	NSC 319990	–13.3	
2	NSC 80731	–13.3	
3	NSC 80735	–13.3	
4	NSC 67436	–13.1	
5	NSC 37553	–13.0	
6	RA-0002034	–9.9	–9.27 [15]

a The unit of energy is kcal mol^–1^.

b The experimental binding free energy was calculated via formula Δ*G*_EXP_ =  *RT*ln(*K*_i_), where *R* is the gas constant, *T* is the absolute temperature, and *K*_i_ is the inhibition constant.

### 3.2. Refining docking results with molecular dynamics simulations

It should be noted that molecular docking simulations commonly use several approximations and constraints to speed up the calculation process [59–61]. The applied simplifications involved using a rigid receptor, using an implicit solvent model, incorporating a united atom model limiting a number of trail binding pose, etc. [35,60,62,63]. Therefore, although the molecular docking simulations provided an appropriate outcome, MD simulations were then carried out to refine the results. The complexes, including CHIKV nsP2pro + NCI compounds, were parametrized and mimicked using MD simulations. Besides, the CHIKV nsP2pro + RA-0002034 was also investigated because RA-0002034 acts as a positive control. The ligand binding pose and nsP2pro structure changed during MD simulations (cf. figures S1–S6 in the supplementary material). In particular, ligands have established highly optimized and stable networks of HBs and hydrophobic interactions with important active-site residues such as Tyr544, Trp549, Asn547 and Ala511. Thus the binding mechanisms were explored based on structural analyses. Each complex was simulated three times in MD simulations with a length of 200 ns. Furthermore, the free ligand in solution was mimicked for over 20 ns of MD simulations. The binding affinity of ligands to CHIKV nsP2pro was clarified using binding free energy protocols [64,65]. Here, perturbation simulations were acquired to complete this task since providing the most accurate results until the date [66,67]. Notably, this method has a proven track record of successful application across numerous biological targets [45,46,68,69,70].

The CHIKV nsP2pro + ligand complexes in solution mostly reached equilibrium conditions after *ca* 100 ns of MD simulations according to the obtained RMSD presented in table S1 in the supplementary material. Moreover, the free ligand in solution also reached stable states after *ca* 2.5 ns of MD simulations (cf. supplementary material, table S2). The systemic coordinates within the equilibrium intervals were collected for structural analysis. To clarify the binding picture between nsP2pro and inhibitor, the representative conformations of the complexes in solution were estimated using the clustering method over all equilibrium snapshots. A non-hydrogen atom cut-off of 0.3 nm was applied. The ligand interaction diagrams were then investigated using PLIP web application [54]. The outcomes are described in figure 2 and figures S1–S5 in the supplementary material. MD-refined findings suggested that the residues Asn476, Ala511, Tyr512, Tyr544 and Trp549 act as crucial residues controlling the binding process of nsP2pro inhibitors. Although top-leads and the available inhibitor do not form rigid interactions to Cys478 or Ser482 or His548, the six compounds can change the pairs involving Cys478-Sγ–Nε-His548 and Ser482-Oγ–Nε-His548 via interacting with Asn476 and Trp549. Indeed, the pairs including Cys478-Sγ–Nε-His548 and Ser482-Oγ–Nε-His548 were significantly changed when the ligands were induced. The outcomes are described in figure 3 and table S3 in the supplementary material. In particular, the average of the spacing between Cys478-Sγ–Nε-His548 and Ser482-Oγ–Nε-His548 is 7.4 ± 1.4 and 8.9 ± 1.8 Å, respectively, when the free nsP2pro in solution is considered. When the considered inhibitors were induced, the distances of the pair Cys478-Sγ – Nε-His548 are significantly enlarged compared to the ones of the free nsP2pro in solution and nsP2pro + RA-0002034 systems (cf. supplementary material, table S3). Meanwhile, the distance of the pair Ser482-Oγ - Nε-His548 varies depending on the specific ligand (cf. Table S3 of the Supporting Information). The findings suggest that five NCI compounds can prevent the biological activity of nsP2pro.

**Figure 3.**
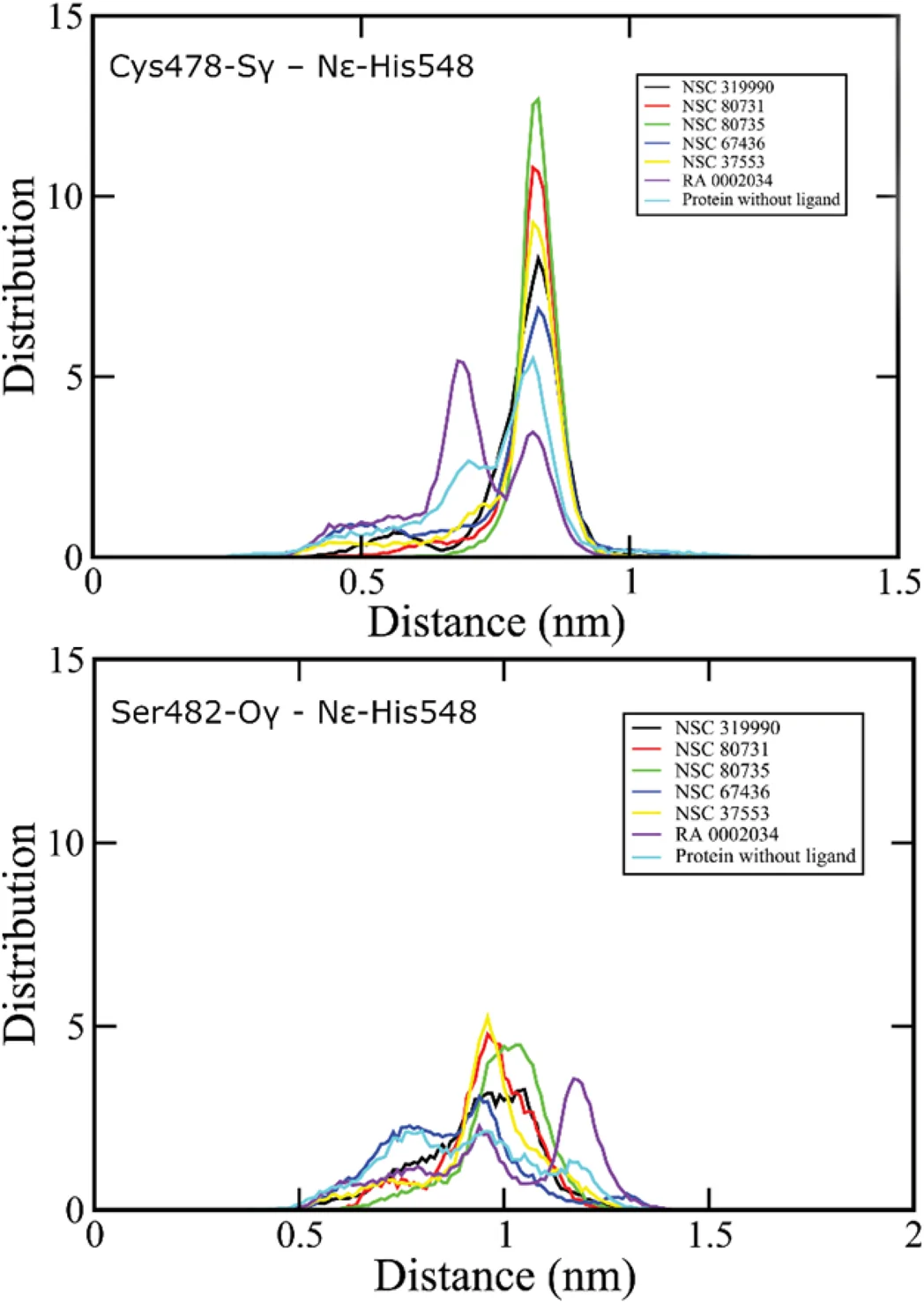
Distribution of the pairs between Cys478-Sγ–Nε-His548 (upper panel) and Ser482-Oγ–Nε-His548 (lower panel). The results are obtained by analysing the MD-refined equilibrium snapshots.

### 3.3. Perturbation simulations

The binding free energy of ligands to CHIKV nsP2pro can be accurately estimated via FEP simulations. During simulations, the coupling parameter λ was changed to turn the system from *bound* to *unbound* states. The free energy alteration of the nsP2pro + inhibitor and free inhibitor in solution was computed using λ-alteration simulation. In particular, alchemical free energy profiles of coulomb, vdW and total terms over MD simulations are shown in figure S7 in the supplementary material. Moreover, the smooth Δ*G* versus *λ* transitions along with small computed error bars reveal excellent phase-space overlap, proving that the number of applied *λ* windows is sufficient (cf. supplementary material, figure S8). Furthermore, the free energy difference between two processes is calculated as the binding free energy. The obtained results are described in table 2. The binding free energy of RA-0002034 to nsP2pro via FEP calculations is Δ*G*_FEP_ = −6.45 ± 0.32 kcal mol^–1^, which is significantly smaller than the experimental value, Δ*G*_EXP_ = −9.27 kcal mol^–1^. The difference may be owing to the fact that only non-covalent binding free energy is taken into account in the calculations. Moreover, all five NCI compounds exhibited significantly larger binding affinities to CHIKV nsP2pro than the available inhibitor named RA-0002034. In particular, the NSC 80731 and NSC 37553 bind to nsP2pro with the strongest binding free energy of –11.71 ± 1.25 and –11.14 ± 0.32 kcal mol^–1^, respectively. The others including NSC 80735, NSC 67436 and NSC 319990 also unveiled a stronger binding affinity than RA-0002034 with corresponding values of –10.91 ± 2.00, –10.13 ± 0.73 and –9.70 ± 0.61 kcal mol^–1^, respectively. Five NCI compounds act as highly potential inhibitors as compared with the CHIKV nsP2pro.

**Table 2.** The ligand binding affinity provided by perturbation simulations.^a^

no.	ligand	Δ*G*_cou_	Δ*G*_vdW_	Δ*G*_FEP_	Δ*G*_EXP_^b^
1	NSC 319990	–0.94	–8.75	–9.70 ± 0.61	
2	NSC 80731	–0.51	–11.20	–11.71 ± 1.25	
3	NSC 80735	0.03	–10.94	–10.91 ± 2.00	
4	NSC 67436	–0.68	–9.45	–10.13 ± 0.73	
5	NSC 37553	–0.46	–10.68	–11.14 ± 0.32	
6	RA-0002034	1.43	–7.88	–6.45 ± 0.32	–9.27 [15]

a The unit of energy is kcal mol^–1^.

b The experimental binding free energy was calculated via formula Δ*G*_EXP_ =  *RT*ln(*k*_i_), where *R* is the gas constant, *T* is the absolute temperature, and *k*_i_ is the inhibition constant.

A good correlation, *R* = 0.92, between docking and FEP calculations was obtained once again, confirming the concordance of using mVina for screening nsP2pro inhibitors. In addition, the strong correlation, *R* = −0.92, between the binding free energy, Δ*G*_FEP_, and the distance Cys478-Sγ–Nε-His548 suggests that stronger binding ligand better inhibits the biological activity of the CHIKV nsP2pro.

### 3.4. Absorption, distribution, metabolism and excretion estimations

To evaluate the pharmacokinetic and drug-likeness properties of the proposed inhibitors, ADME calculations were performed using pkCSM [55]. Detailed data are reported in table S4 in the supplementary material. In particular, all candidate compounds exhibited significantly higher human intestinal absorption (greater than 82%), compared with the reference inhibitor RA-0002034 (69%). These compounds also show well-regulated total clearance rates and present no skin sensitization risks. Moreover, the top-lead compounds, NSC 319990 and NSC 80731, successfully passed the AMES toxicity test, indicating no mutagenic potential. Although these high-affinity inhibitors violate Lipinski's Rule of Five owing to their relatively high molecular weight and lipophilicity, such violations are common among typical antiviral protease inhibitors. Consequently, these identified compounds represent highly promising structural scaffolds for further lead optimization.

## 4. Conclusion

In this work, the possible inhibitors for CHIKV nsP2pro were investigated using computational works. In particular, the ligand binding process to nsP2pro, an important enzyme for viral replication, was intensively investigated by combining molecular docking, molecular dynamics and perturbation simulations.

The outcomes indicated that five NCI compounds could act as possible agents to inhibit CHIKV nsP2pro. These inhibitors exhibit rigid, non-covalent binding affinities to the viral protease that are significantly larger than those of the available inhibitor RA-0002034. Moreover, MD-refined simulations were then carried out to clarify the binding poses and factors of ligands to nsP2pro. During the equilibrium intervals, the ligands rigidly formed contacts with the residues Asn476, Ala511, Tyr512, Tyr544 and Trp549 of nsP2pro. Furthermore, five NCI compounds probably prevent the biological activity of CHIKV nsP2pro by increasing the distance of the pairs Cys478-Sγ–Nε-His548 and Ser482-Oγ–Nε-His548. In addition, ADME estimation confirmed that these top candidates possess excellent human intestinal absorption metrics, identifying them as druggable lead scaffolds and safe.

Overall, the findings suggested that five NCI compounds are highly promising candidates for further experimental validation as novel CHIKV nsP2pro inhibitors. However, these findings are based on *in silico* models; therefore, future *in vitro* enzymatic and cell-based antiviral assays are fundamentally required to validate the obtained results.

## Supplementary Material



## Data Availability

Initial conformation/file of molecular docking and MD simulations can be downloaded from [71]. Supplementary material is available online [72].
