# Supplementary material for: Computational identification of potential inhibitors for chikungunya virus nsP2pro
Source: R Soc Open Sci. 2026 Oct 7;13(10):252347. doi: 10.1098/rsos.252347 (PMC13641887; doi:10.1098/rsos.252347)
Supplement: Supplementary file 1 [file rsos.252347.zip › rsos.252347/rsos252347_si_001.pdf]

Supporting information

Computational Identification of Potential Inhibitors for Chikungunya Virus nsP2pro

Hoang Anh Nguyen,[a] Son Tung Ngo[bc*]

[a]*NTT Hi-Tech Institute, Nguyen Tat Thanh University, Ho Chi Minh City 72820, Vietnam*

[a]*Laboratory of Biophysics, Institute of Advanced Study in Technology, Ton Duc Thang University, Ho Chi Minh City 72915, Vietnam*

[b]*Faculty of Pharmacy, Ton Duc Thang University, Ho Chi Minh City 72915, Vietnam*

Email: ngosontung@tdtu.edu.vn

Table S1. RMSD of 6 complexes including nsP2pro + ligands and RMSD of the free nsP2pro in solution.

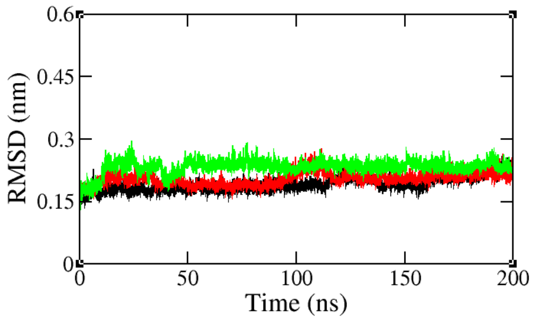

nsP2pro + NSC 319990

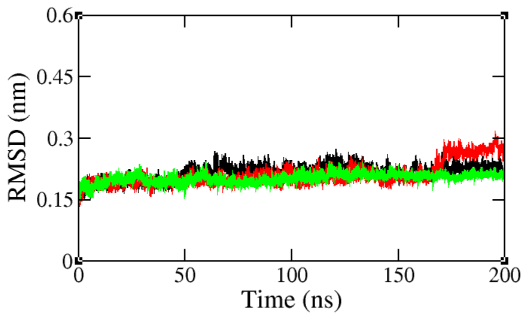

nsP2pro + NSC 80731

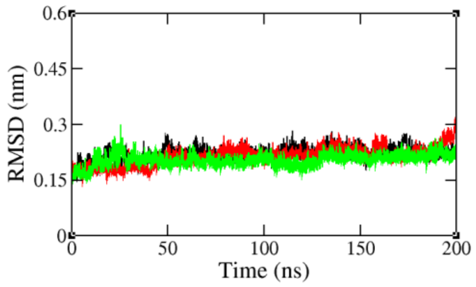

nsP2pro + NSC 80735

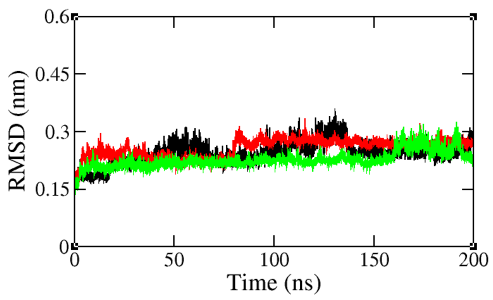

nsP2pro + NSC 67436

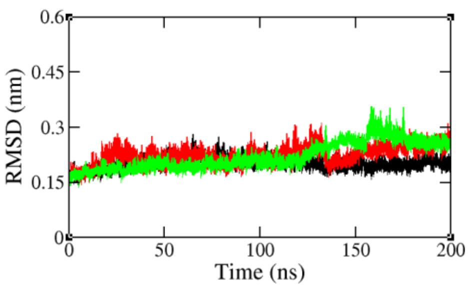

nsP2pro + NSC 37553

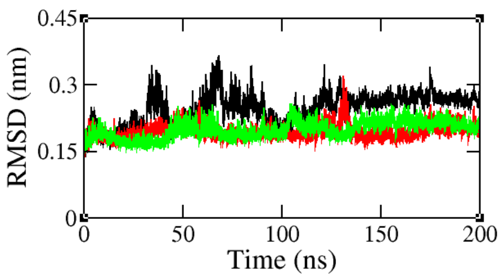

nsP2pro + RA-0002034

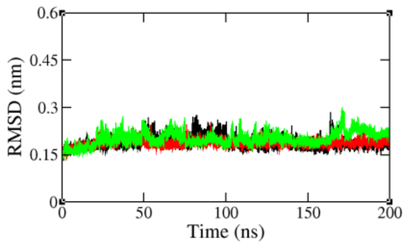

Free nsP2pro in solution

Table S2. RMSD of 6 free ligands in solution.

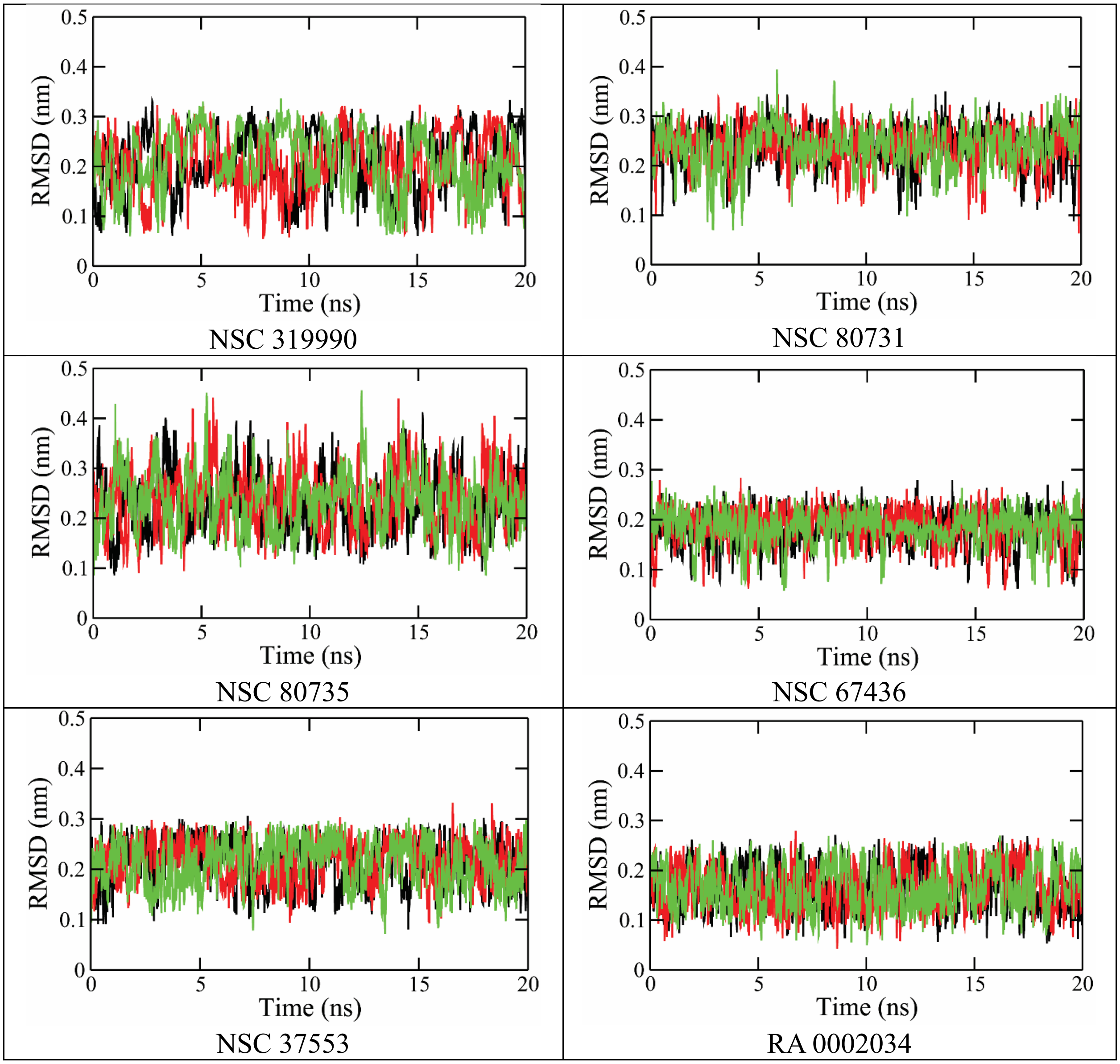

Table S3. The average of spacing between two atoms including Cys478-Sγ – Nε-His548 and Ser482-Oγ - Nε-His548[a]

System	Cys478-Sγ – Nε-His548	Ser482-Oγ - Nε-His548
nsP2pro + NSC 319990	7.9 ± 0.9	9.3 ± 1.2
nsP2pro + NSC 80731	8.1 ± 0.7	9.6 ± 1.1
nsP2pro + NSC 80735	8.3 ± 0.4	9.9 ± 1.0
nsP2pro + NSC 67436	7.7 ± 1.4	8.7 ± 1.6
nsP2pro + NSC 37553	7.9 ± 1.1	9.5 ± 1.4
nsP2pro + RA-0002034	6.9 ± 1.2	9.9 ± 2.1
nsP2pro without ligand	7.4 ± 1.4	8.9 ± 1.8

[a]The unit is of Angstrom.

Table S4. Predicted ADME of six ligands.

	NSC 319990	NSC 80731	NSC 80735	NSC 67436	NSC 37553	RA 0002034
ABSORPTION						
Water solubility	-3.56	-3.97	-3.31	-4.22	-3.69	-3.31
Caco2 (cm s^{-1})	0.26	0.06	-0.30	0.62	0.64	-0.16
HIA (%)	99.37	97.37	88.83	82.57	90.43	69.00
Skin Permeability (log Kp)	-2.74	-2.74	-2.74	-2.74	-2.74	-2.90
P-glycoprotein substrate	Yes	Yes	Yes	Yes	Yes	Yes
P-glycoprotein I inhibitor	No	No	Yes	Yes	Yes	No
P-glycoprotein II inhibitor	Yes	Yes	Yes	Yes	Yes	No
METABOLISM						
CYP2D6 substrate	No	No	No	No	Yes	No
CYP3A4_substrate (PRE)	Yes	Yes	Yes	Yes	Yes	No
CYP1A2 inhibitor	No	No	No	No	Yes	No
CYP3A4_inhibitor (PRE)	Yes	Yes	Yes	Yes	Yes	No
CYP2D6_inhibitor (PRE)	No	No	No	No	No	No
CYP2C9_inhibitor (PRE)	No	Yes	Yes	Yes	Yes	No
CYP2C19_inhibitor (PRE)	Yes	Yes	Yes	Yes	Yes	No
EXCRETION						
Total Clearance	-0.14	0.36	-0.01	-0.14	0.36	0.72
Renal OCT2 substrate	No	No	No	No	No	No
TOXICITY						
AMES	No	No	Yes	Yes	Yes	No
hERG I inhibitor	No	No	No	No	No	No
hERG II inhibitor	Yes	Yes	Yes	Yes	Yes	Yes
Oral Rat Acute Toxicity	2.68	2.51	2.44	2.26	2.71	2.78
Oral Rat Chronic Toxicity	2.21	0.89	4.01	2.54	2.09	1.96
Hepatotoxicity	Yes	Yes	Yes	Yes	Yes	Yes
Skin Sensitisation	No	No	No	No	No	No
DISTRIBUTION						
VDss (human, log L/kg)	-0.29	-1.16	-1.71	-0.10	-0.32	-0.51
Fraction unbound	0.00	0.00	0.07	0.00	0.16	0.30
Log BBB	-1.28	-1.23	-1.47	-0.80	-0.11	-1.60
CNS permeability (log PS)	- 1.65	-1.27	-1.87	-2.43	-1.28	-3.84
PROPERTIES						
Weight	474.57	507.38	528.48	486.96	476.58	349.41
LogP	6.5024	8.07	6.58	3.54	6.40	1.76
Acceptors	6	3	7	6	2	5
Donors	4	4	4	4	2	2
TPSA ($Å^2$)	196.25	211.58	220.28	207.07	210.73	140.20
Rule of Five	Violated	Violated	Violated	Violated	Violated	Suitable

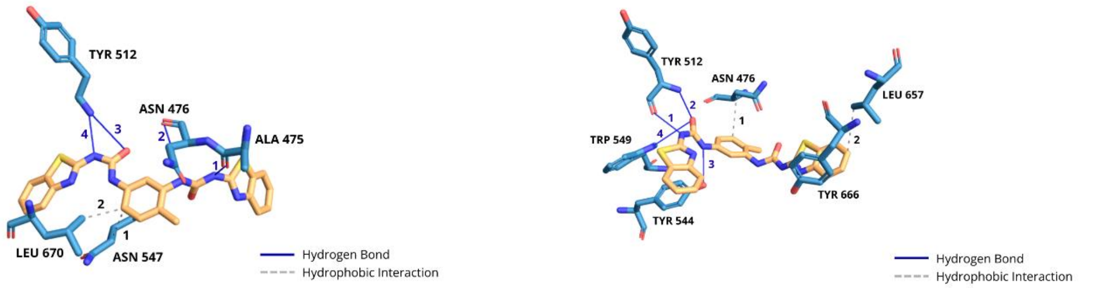

(A) (B)

Figure S1. Interaction diagram between CHIKV nsP2pro and NSC 319990 in docking mode (A) and MD refined structure (B), which was prepared via PLIP webapp.[1]

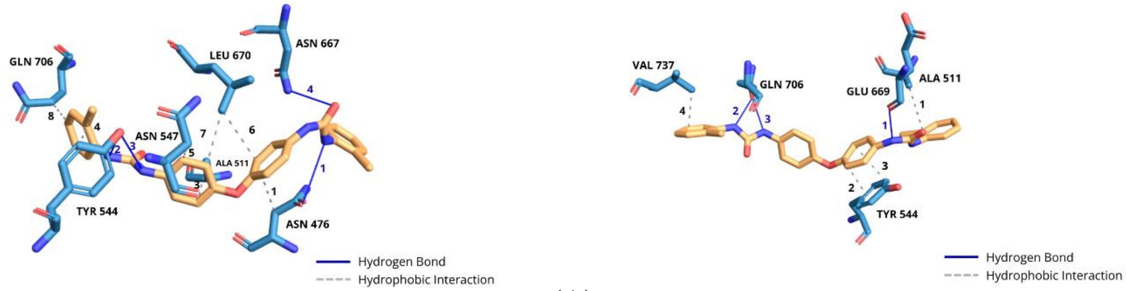

(A) (B)

Figure S2. Interaction diagram between CHIKV nsP2pro and NSC 80731 in docking mode (A) and MD refined structure (B), which was prepared via PLIP webapp.[1]

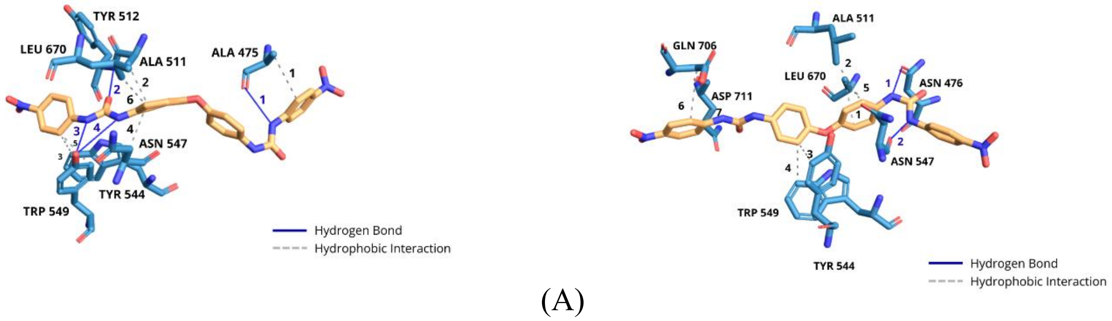

(A) (B)

Figure S3. Interaction diagram between CHIKV nsP2pro and NSC 80735 in docking mode (A) and MD refined structure (B), which was prepared via PLIP webapp.[1]

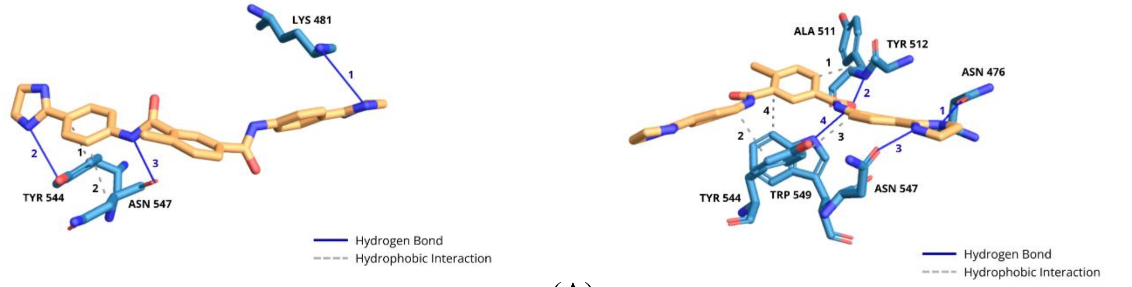

(A) (B)

Figure S4. Interaction diagram between CHIKV nsP2pro and NSC 67436 in docking mode (A) and MD refined structure (B), which was prepared via PLIP webapp.[1]

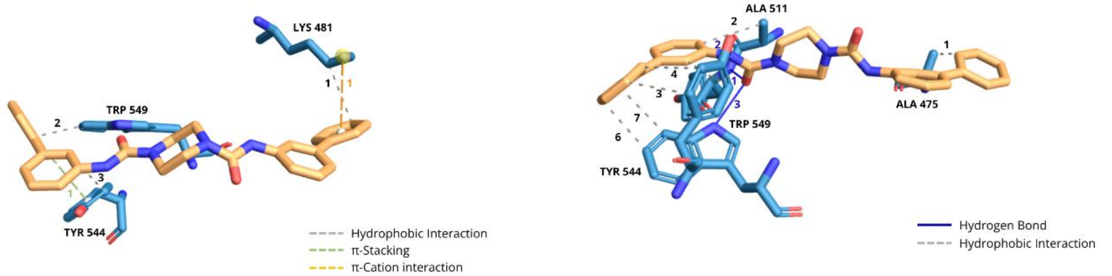

(A) (B)

Figure S5. Interaction diagram between CHIKV nsP2pro and NSC 37553 in docking mode (A) and MD refined structure (B), which was prepared via PLIP webapp.[1]

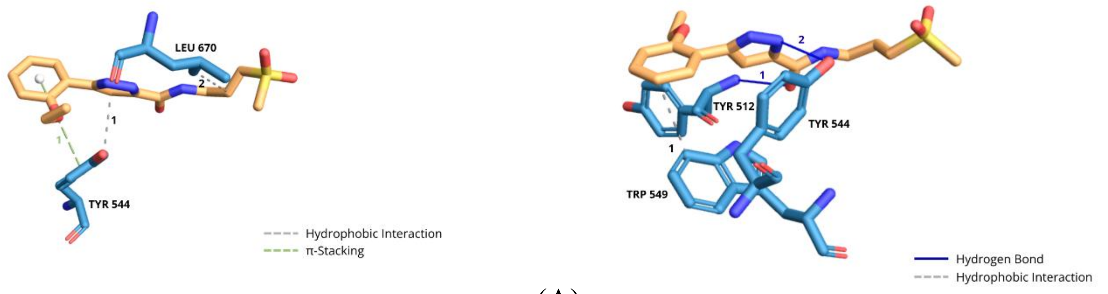

(A) (B)

Figure S6. Interaction diagram between CHIKV nsP2pro and RA-0002034 in docking mode (A) and MD refined structure (B), which was prepared via PLIP webapp.[1]

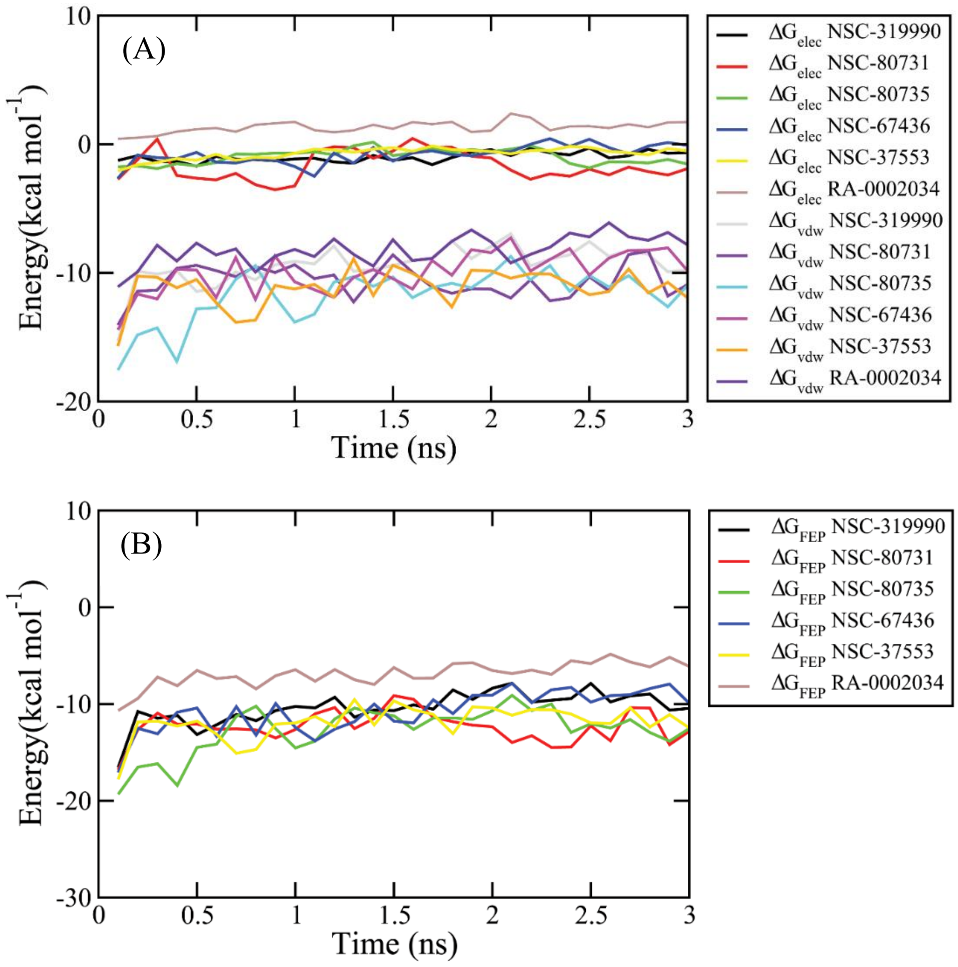

Figure S7. Alchemical free energy profiles for the six complexes as a function of simulation time. (A) Contributions of the free electrostatic and vdW energy components. (B) Evolution of the total alchemical binding free energy over the 3 ns simulation window.

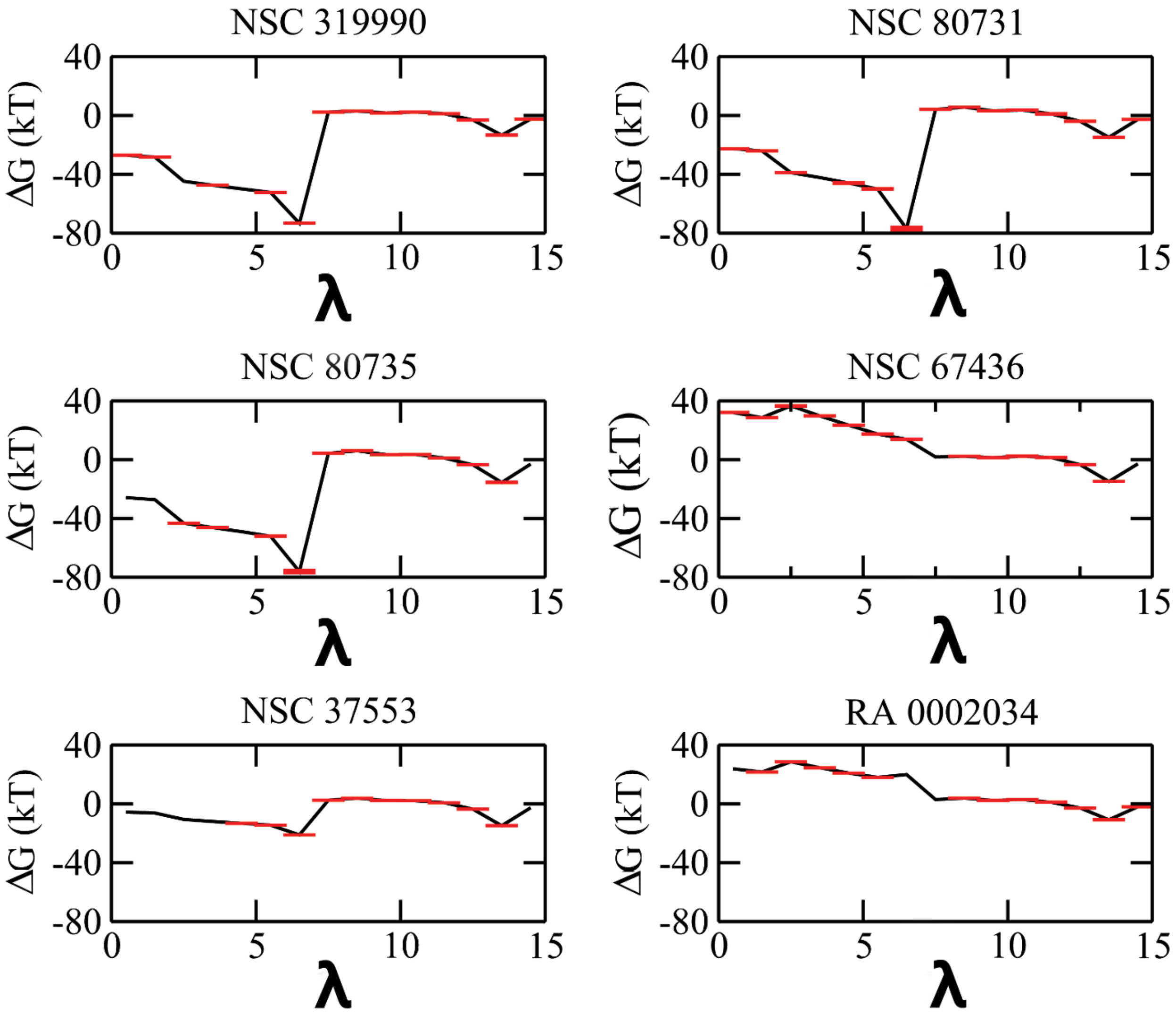

Figure S8. Alchemical binding free energy profiles across the intermediate λ states for the six complexes. The red horizontal segments denote the computed errors for each alchemical window, demonstrating smooth structural transitions and excellent phase-space overlap.
